# Measuring child food poverty: understanding the gap to achieving minimum dietary diversity

**DOI:** 10.1017/S1368980025000023

**Published:** 2025-01-08

**Authors:** Sebastian Vollmer, Arnaud Laillou, Nora Albers, Simeon Nanama

**Affiliations:** 1 University of Goettingen, Waldweg 26, Göttingen 37073, Germany; 2 UNICEF West and Central Africa Regional Office, Immeuble Madjiguène, Route des Almadies. PO Box 29720, Dakar, Senegal

**Keywords:** Complementary feeding, Food, Africa, MDD, Minimum dietary diversity, Eggs

## Abstract

**Objective:**

The aim of this study is to analyse complementary feeding practices, to assess the extent to which minimum dietary diversity (MDD) recommendations are being met in the population studied and to study factors that influence the achievement of MDD.

**Design:**

We pooled individual level data form the Demographic and Health Surveys (DHS) and Multi Indicator Cluster Surveys (MICS). We apply methods from poverty measurement to identify individual gaps towards achieving MDD. We further identify food groups that separate children who achieve MDD from those who do not.

**Setting:**

West and Central Africa.

**Participants:**

62 257 children aged between 6 and 23 months.

**Results:**

82·0 per cent of children do not achieve MDD and on average are lacking 2·5 out of five required food groups. For 19·0 per cent of children, the gap to MDD is one food group and for 23·7 per cent of children the gap is two food groups. Consumption of eggs, other fruits and vegetables as well as legumes and nuts is particularly low among children who are not achieving MDD. More than 90·0 per cent of children who do not achieve MDD do not consume these food groups compared to around half of children who achieve MDD.

**Conclusions:**

Overall MDD is low, but there is large potential for improving MDD achievement if food consumption can be increased by one or two food groups. Available, affordable and culturally accepted food groups are identified that could be prioritised in interventions to close this gap.

The WHO recommends exclusive breast-feeding for the first 6 months of a baby’s life and to complement breastmilk with other foods between 6 and 23 months of age to ensure sufficient micronutrient density that is needed for both cognitive and physical development^(1,2)^. Minimum dietary diversity (MDD) is one of the indicators aimed at assessing the quality of these complementary foods in terms of nutrient intake for children of this age group. MDD as defined by WHO is achieved if a child consumes at least five out of eight distinct food groups over a period of 24 h. If a child fails to achieve the consumption of at least five out of eight food groups, it is considered to suffer from food poverty^(3)^.

There is a large number of studies that investigate the association of MDD with different socio-economic factors^(4–15)^. These studies document that higher household wealth, higher parental education, lower number of siblings, maternal knowledge, use of antenatal care or media exposure are positively correlated with children achieving MDD. However, all cited studies treated MDD as a binary outcome. They did not explicitly study the number of food groups consumed or consumption patterns of the specific underlying food groups to develop interventions to improve MDD.

Several studies discussed the consumption of certain food groups in the context of MDD, for instance, by mentioning which food groups were mostly consumed by children who do not meet MDD^(16–20)^. There are two other studies that compared food group consumption patterns between children meeting and not meeting MDD. Beckerman-Hsu et al. (2020) describe such differences for children from India and Heemann et al. (2022) for children from fifty-nine low- and middle-income countries. However, these studies also treated MDD as a binary concept, and none of the before mentioned papers quantified the gap to achieving MDD^(21,22)^.

The aim of this study is to develop a more comprehensive understanding of dietary diversity in children, moving beyond the binary view of meeting *v*. not meeting MDD. In consequence, we want to better understand the extent of child food poverty, to improve guidance and support for vulnerable children, and to better track progress towards nutrition goals. For this purpose, we propose to adapt the well-known approach of Foster, Greer and Thorbecke (1984) for poverty measurement to the measurement of absence of MDD^(23)^. We interpret absence of MDD as poverty in the quality of children’s diet and the threshold of five food groups as analogy to the poverty line.

This allows us, in addition to measuring the share of children who do not achieve MDD, to quantify their MDD gap, that is, the percentage of food groups missing for achieving MDD. A specific objective of this study is to compare individual food group consumption patterns for children meeting and not meeting MDD specifically for children who fall just one or two food groups short of achieving MDD. This comparison allows us to highlight specific food groups that, if added to the diet, could significantly improve the dietary diversity of a large proportion of children. We further discuss availability and affordability of different food items in the food groups as well as cultural barriers to their consumption.

## Methods

### Data source

We pooled Demographic and Health Surveys (DHS) and Multi Indicator Cluster Surveys (MICS) for West and Central African countries. Both DHS and MICS are nationally representative surveys that include detailed information on children’s food intake.

### Study setting

The sample design of the two data sources may differ slightly, especially in terms of age range and geographic coverage, as the MICS data also provide subnational information. However, these differences are negligible because the age range was restricted to children 6–23 months and the analysis is ascertained on a national basis. The age group 6–23 months was chosen according to the WHO definition of MDD, as dietary diversity across food groups at this age is particularly important for cognitive and physical development. Both DHS and MICS aim to provide comparable data across countries and over time. The standardised methods and protocols allow for comparison of data across countries and facilitate tracking of trends over the years. Thus, in order to use the most recent data possible from developing countries in West and Central Africa, this paper combines the two data sources from DHS and MICS (compare Corsi et al. (2017)^(24)^).

For each country, we took the most recent available survey which is either DHS or MICS. This also ensures that the individuals are mutually exclusive. Data were available for twenty-two out of the twenty-four countries of the UNICEF West and Central Africa region. The surveys were conducted between 2010 and 2020. No data were available for Cabo Verde and Equatorial Guinea. The pooled sample included 62 257 children between 6 and 23 months of age with non-missing observations for dietary diversity (Table 1). 4157 children between 6 and 23 months of age had missing observations for dietary diversity.

**Table 1.** MDD-FGT measures by country

	Year	*n*	MDD-FGT_0_ percent	MDD-FGT_1_ percent	Missing FG
Benin	2018	3868	75·3	39·8	2·8
Burkina Faso	2010	2065	95·4	51·6	2·8
Cameroon	2018	2549	82·5	36·8	2·2
Central African Republic	2018	2516	89·8	47·2	2·6
Chad	2019	5422	76·3	39·8	2·6
Congo	2014	2765	87·6	41·0	2·3
Democratic Republic of Congo	2017	6499	85·0	37·8	2·2
Gabon	2012	1176	86·7	41·2	2·4
Gambia	2020	2302	81·6	37·5	2·3
Ghana	2017	2575	77·4	36·5	2·4
Guinea	2018	1898	86·7	49·0	2·8
Guinea-Bissau	2018	2193	93·3	50·6	2·7
Ivory Coast	2016	2668	82·5	38·2	2·3
Liberia	2020	1507	91·4	44·6	2·4
Mali	2018	2704	78·8	41·9	2·7
Mauritania	2020	3061	79·9	39·2	2·5
Niger	2012	1559	88·7	49·4	2·8
Nigeria	2018	8575	77·7	36·4	2·3
Sao Tome and Principe	2019	503	69·8	29·5	2·1
Senegal	2019	1760	82·2	39·3	2·4
Sierra Leone	2019	2632	73·3	35·0	2·4
Togo	2017	1460	82·0	35·5	2·2
Total		62 257	82·0	40·4	2·5

MDD, minimum dietary diversity; FGT, Foster-Greer-Thorbecke; FG, food groups.

Information on children’s food intake were collected based on a 24-h food consumption-recall of the mothers. The surveys include information on the consumption of specific standardised food items that were grouped into eight food groups as follows:

1. Grains, roots and tubers: Commercially fortified cereal (baby food); Bread, rice, noodles or foods made from grains; White potatoes, white yams, manioc, cassava or any other foods made from roots
2. Legumes and nuts: Beans, peas, lentils or nuts
3. Dairy products: Powdered, tinned milk or fresh animal milk; Infant formula; Yogurt; Cheese or other milk products
4. Flesh foods: Any meat (beef, pork, lamb, goat, chicken or duck); Liver, heart, other organ meats; Fresh or dried fish or shellfish
5. Eggs: Eggs
6. Vitamin A-rich fruits and vegetables: Pumpkin, carrots, squash or sweet potatoes; Any dark green leafy vegetables; Ripe mangoes, papayas, other vitamin A fruit
7. Any other fruits and vegetables: Other fruits or vegetables
8. Breastmilk: Currently breastfed

### Outcome variables

MDD is defined as a binary variable that is assigned the value 1 if a child consumed at least five out of the eight food groups in the previous 24 h. We propose to study absence of MDD within the framework of poverty measurement using the commonly used Foster–Greer–Thorbecke (FGT) poverty measure:

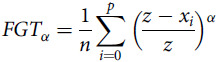

with *z* as poverty line, *x*_*i*_ as income of individual *i*, *n* as number of people and *p* as number of people below the poverty line. In the context of MDD, the analogy to the poverty line is the consumption of at least five food groups (z) and the analogy to the income *x*_*i*_ is the number of food groups consumed by child *i*. *p* is the number of children who consume less than five food groups and *n* is the total number of children.

*α* denotes a fixed parameter that in the FGT poverty measure usually takes on values *α* = 0, 1 and 2. For *α* = 0, the formula reduces to *p/n* and is called poverty headcount ratio, the share of individuals who are living in poverty. In the context of MDD, this would be the share of children who do not achieve MDD. For *α* = 1, the FGT measure also takes into account the distance of the poor from the poverty line and measures the percentage gap (*z − x*_*i*_)*/z* of poor individuals from the poverty line. For non-poor people, this income gap is zero. In the context of MDD, this would mean that a child that consumes four food groups is missing 20 per cent of the five required food groups to achieve MDD, a child who consumes three food groups is missing 40 per cent, and so on. Children who achieve MDD have a gap of 0 per cent. The FGT poverty measure for *α* = 1 is called poverty gap and can be expressed as the product of the headcount ratio and the average gap (in percent) of people below the poverty line. It quantifies the overall distance of those below the poverty line from the poverty line. The poverty gap provides important information beyond the simple headcount ratio. For instance, to track progress towards achieving SDG 1 ‘No Poverty’, it is not only relevant to know how many people are poor but also to what extent the poor are getting closer to the poverty line and thus how much more progress is needed to lift them out of poverty. We argue that the same is also true for SDG 2 ‘Zero Hunger’ and our measure of MDD child food poverty. It is not only relevant to know how many children do not achieve MDD but also what fraction of food groups they are missing. FGT poverty measures with *α ≥* 2 also measure a form of the poverty gap but give larger weight to those individuals who are further away from the poverty line. For larger *α*, the FGT measures lose their simple numerical interpretation as share (in case of FGT_0_) or distance (in case of FGT_1_). While FGT_2_ also measures a gap due to the squaring it is no longer measured in the same units as the original variable and therefore more difficult to interpret. For the ease of quantitative interpretation, we focus on FGT measures with *α* = 0 and *α* = 1 and will refer to them as MDD poverty rate (MDD-FGT_0_) and MDD poverty gap (MDD-FGT_1_).

In addition to the MDD-FGT, we also present consumption patterns by food groups, separately for children meeting and not meeting MDD as well as specifically for children who are just one or two food groups away from achieving MDD. This analysis can help to guide appropriate interventions in the right direction such that more children in West and Central Africa reach MDD.

To better understand the underlying reasons for the low consumption of certain food groups, we discussed the regional- and country-level results with representatives from the UNICEF country offices of the West and Central Africa region. The meetings were conducted in five groups that were organised according to ecological zone: (1) Sahel (Mali, Burkina Faso, Niger, Mauritania and Chad), (2) Coast 1 (Senegal, Gambia, Guinea Bissau and Guinea), (3) Coast 2 (Ivory Coast, Benin, Ghana, Togo, Liberia and Sierra Leone), (4) Coast 3 (Nigeria, Cameroon, Gabon and Sao Tome) and (5) Central Africa (Central African Republic, Congo Democratic Republic and Congo). The same key questions on accessibility and barriers were consistently used in our Focus Group Discussions, which can be found in the appendix. Findings from these conversations with local nutrition experts are included in the discussion section.

## Results

**Figure. 1:**
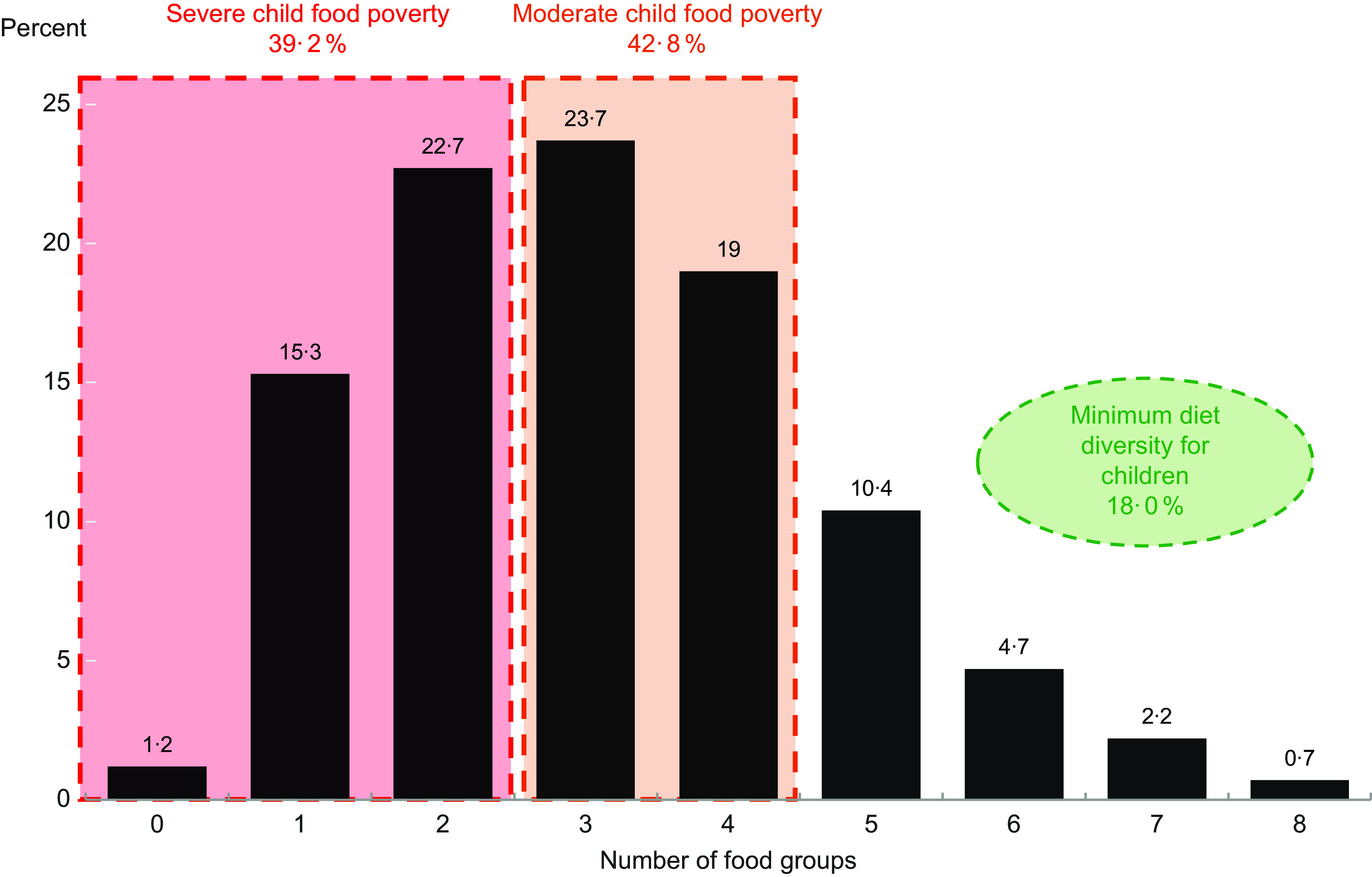
Number of food groups consumed by children (6–23 months).

Figure 1 shows the share of children in West and Central Africa per number of consumed food groups. Overall, 18·0 per cent of all children meet the MDD by consuming five or more food groups. Another 19·0 per cent consume exactly four food groups, needing just one more to meet the MDD. If these children achieved MDD, the proportion meeting the criteria would more than double. In addition, 23·7 per cent of children consume exactly three food groups and need two more to reach MDD. If these children also achieve MDD, the share of children meeting MDD would more than triple. This breakdown by number of food groups shows that despite the very high levels of absence of MDD, there is a large potential for improvement because many children are quite close to the threshold of five food groups. Results by country and by ecological zone are shown in the Appendix.

**Figure. 2:**
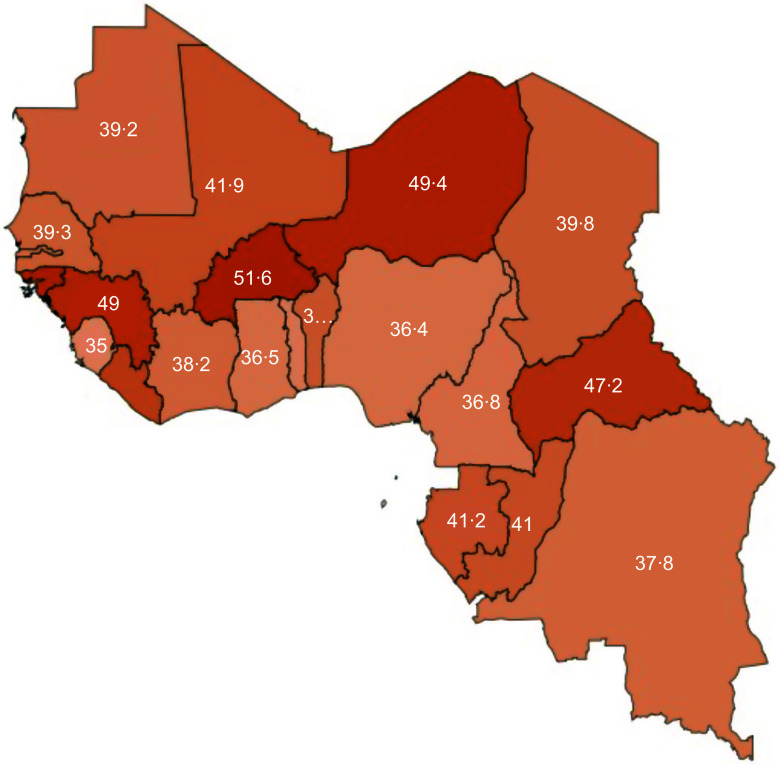
MDD poverty gap (MDD-FGT1) across WCAF countries (Excluding Equatorial Guinea and Capo Verde). MDD, minimum dietary diversity.

Table 1 shows for each country and the pooled sample the MDD-FGT measures and the average number of food groups missing for children who do not achieve MDD. In Burkina Faso, the MDD poverty rate (i.e. MDD-FGT_0_) is 95·4 per cent of children, the MDD poverty gap (i.e. MDD-FGT_1_) is 51·6 per cent and MDD poor children on average consume 2·2 food groups and are thus lacking 2·8 food groups. In Sao Tome and Principe, the MDD poverty rate is 69·8 per cent of children, the MDD poverty gap is 29·5 per cent and MDD poor children on average are lacking 2·1 food groups. For the pooled sample of twenty-two countries, the MDD poverty rate is 82·0 per cent of children, the MDD poverty gap is 40·4 per cent (Fig. 2) and MDD poor children on average are lacking 2·5 food groups.

The MDD poverty gap (MDD-FGT_1_) can be derived by multiplying the MDD poverty rate (MDD-FGT_0_) with the share of food groups that people in poverty on average are missing. We can therefore have very different MDD poverty gaps for similar levels of MDD poverty rates. For instance, if all people below the poverty line are quite close to the poverty line, the MDD poverty gap will be close to zero. In contrast, if all people below the poverty line are close to zero, then the MDD poverty gap is close to the MDD poverty rate. We find such differences, although not as extreme as in this illustrative example when we compare countries with similar MDD poverty rates. For Cameroon, DRC and Togo, the MDD poverty rate is relatively high, but the MDD poverty gap is much smaller than for other countries with similar levels of MDD poverty rates. This is also reflected in the observations in the sense that children who are MDD poor in these three countries are ‘only’ lacking 2·2 food groups, which is substantially less than in other countries with similar levels of MDD poverty rates. The opposite is true for Benin, Chad and Mali. Their MDD poverty gap is much higher than in other countries with similar, relatively low, levels of MDD poverty rates. In these countries, children who are MDD poor are lacking 2·6–2·8 food groups. Figure 2 shows the MDD poverty gap by country.

**Figure. 3:**
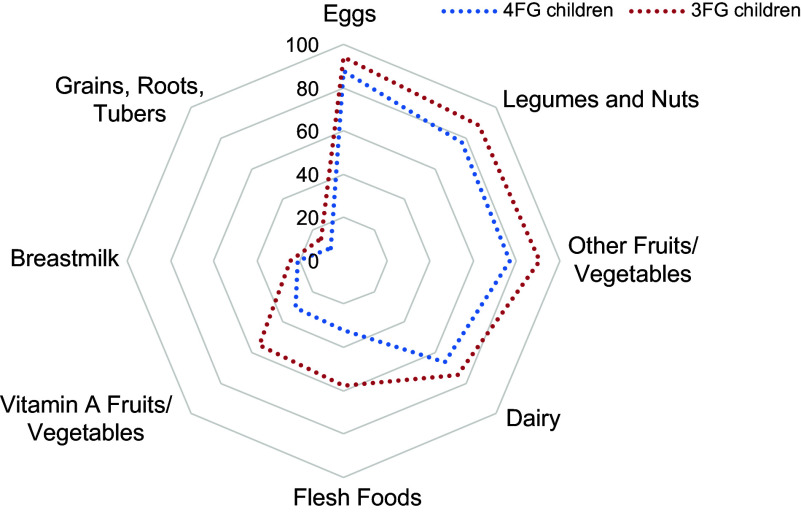
Missing food groups for children who consume three or four food groups.

Table 2 investigates the question which food groups separate children who achieve MDD and those who do not. Specifically, it shows for each food group the share of children in West and Central Africa who do not consume this food group by MDD status. First column: children who achieve MDD, second column: children who do not achieve MDD, third column: children who consume exactly four food groups, fourth column: children who consume exactly three food groups. The difference between children who achieve MDD and those who do not is largest for flesh foods, only 12·6 per cent of children who achieve MDD do not consume flesh foods, whereas this number is 66·3 per cent for children who do not achieve MDD. The difference is similarly large for vitamin A-rich fruits and vegetables. When we focus on children who are already quite close to achieving MDD, that is, children who consume exactly four food groups, the differences are largest for other fruits and vegetables, eggs, dairy products and legumes and nuts. Consumption of eggs, other fruits and vegetables as well as legumes and nuts is particularly low among children who are not achieving MDD with 94·9, 91·1 and 90·3 per cent of children, respectively, not consuming these food groups compared to around half of children who achieve MDD. Breast-feeding is the only food group for which the differences between children who achieve MDD and those who do not are almost zero. For grains, roots and tubers, there is a sizable difference in consumption between children who achieve MDD and those who do not, but this difference is very small when we focus on children who already consume four food groups and are thus close to achieving MDD. Results by country and by ecological zone are shown in the Appendix. For children who consume three or four food groups, the missing food groups are displayed in Fig. 3.

**Table 2.** Missing food groups for children by MDD status (6–23 months)

	MDD	No MDD	4 FG	3 FG
	Percent	Percent	Percent	Percent
Eggs	55·8	94·9	87·5	94·3
	[54·9, 56·7]	[94·7, 95·1]	[86·9, 88·0]	[93·9, 94·6]
Legumes and Nuts	49·0	90·3	77·1	88·5
	[48·0, 49·9]	[90·1, 90·6]	[76·4, 77·9]	[88·0, 89·0]
Other Fruits/Vegetables	42·6	91·1	76·9	90·7
	[41·6, 43·5]	[90·9, 91·4]	[76·1, 77·6]	[90·2, 91·2]
Dairy	37·3	79·7	66·2	74·4
	[36·4, 38·2]	[79·4, 80·1]	[65·4, 67·1]	[73·7, 75·1]
Flesh Foods	12·6	67·9	32·0	57·9
	[12·0, 13·2]	[67·4, 68·3]	[31·2, 32·8]	[57·1, 58·7]
Vitamin A Fruits/Vegetables	15·1	66·3	31·0	55·0
	[14·4, 15·7]	[65·9, 66·7]	[30·2, 31·8]	[54·2, 55·8]
Breastmilk	20·7	23·1	21·2	24·4
	[20·0, 21·5]	[22·7, 23·4]	[20·5, 22·0]	[23·7, 25·1]
Grains, Roots and Tubers	4·0	33·1	8·1	14·8
	[3·6, 4·4]	[32·7, 33·5]	[7·7, 8·6]	[14·2, 15·4]
Observations	11 217	51 040	11 825	14 775

MDD, minimum dietary diversity; FG, food groups.

95 % CI in brackets.

## Discussion

Our analyses and discussions resulted in several salient findings. At first, there is a large share of 82·0 per cent of children in West and Central Africa who do not meet MDD and thus suffer from child food poverty. This is in line with previously documented high levels of children not meeting MDD in low- and middle-income countries. At the same time, the food poverty gap stands at 40·4 per cent, indicating that there are large shares of children, that are not too far off the threshold of achieving MDD in West and Central Africa.

The food groups with the largest missing consumption levels are Eggs, Legumes and Nuts, Other Fruits/Vegetables and Dairy both for children meeting MDD and those children not meeting MDD. The observed low consumption of eggs among children who do not (yet) achieve MDD was a recurring topic in all focus group discussions. The regional nutrition experts point out that eggs are quite expensive in most countries of the West and Central Africa region and that domestic production capacity is limited. Moreover, the egg industry has suffered in recent years due to the avian flu and general shocks to the economy. Limited domestic production and high prices are clearly interlinked and prices would most likely decrease with greater domestic production capacity. The high prices of eggs are also documented by Headey et al. (2018) who compare the prices of different food groups to the cheapest available cereal^(23)^. Whereas eggs are quite cheap in high-income countries, they are very expensive in West and Central Africa with calories from eggs costing 9·9 times more than calories from staple cereals, more than in any other world region.

Another barrier to the demand for eggs are common taboos related to the consumption of eggs. Several experts mentioned in the interviews that eggs are not given to children because of the belief that they will become thieves if they consume eggs. This belief is also documented in the academic literature, for instance, by Ekwochi et al. (2016) and Onuorah and Ayo (2003) for Nigeria^(25,26)^. In a review for a larger set of countries, Iannotti et al. (2014) further document that ‘*cultural beliefs about the digestibility and cleanliness of eggs, as well as environmental concerns arising from hygiene practices and toxin exposures, remain as barriers to widespread egg consumption’* ^(27)^. Flesh foods are the other food group that is quite commonly subject to food taboos^(26,28)^.

An effective strategy to increase the consumption of eggs must therefore address multiple barriers at once. A social and behavioural change communication campaign could inform about the nutritional value of eggs and debunk myths such that children will become thieves if they eat eggs. This alone will not be sufficient though if prices of eggs remain high. Some support of domestic egg production might be needed to reduce prices and local production for own consumption could be strengthened in rural areas. It might be an unrealistic, and also unnecessary, goal to strive for the consumption of one egg per day for every child. Smaller units in form of egg powder are more affordable and possibly also less subject to beliefs about digestibility and cleanliness. Baye et al. (2021) study the affordability of egg powder in the context of Ethiopia and find that with 2·5 g of egg powder/person/day access to the consumption of eggs could be expanded to 1·2 million households (about 4–6 million individuals) in the country^(29)^. Abreha et al. (2021) document the good nutritional values of egg powder and improved properties related to preservability^(30)^. For poorer households, access could also be approved through vouchers or cash transfers. In a recent meta-analysis, Manley et al. (2020) document positive impacts of cash transfers on various nutritional outcomes, including the consumption of animal-sourced foods such as eggs or egg powder^(31)^.

For legumes and nuts, another important food group separating children meeting and not meeting MDD, the story is different. There were no reports about cultural constraints or food taboos related to the consumption of legumes or nuts, and we could also not find any such concerns in the literature. Moreover, legumes and nuts, particularly nuts and beans, are readily available in the region and relatively cheap. However, legumes are considered a cash crop and are difficult to preserve. Promotion and education in the form of community engagement could potentially improve the understanding that a diverse diet including legumes and nuts is very important for young children. In consequence, as this food group is in principle available and affordable in West and Central Africa, it could help to reduce MDD poverty.

Whereas the consumption of vitamin A-rich fruits and vegetables is already relatively high, the consumption of other fruits and vegetables is very low, particularly among children not achieving MDD. According to Headey et al. (2018), calories from vitamin A-rich fruits and vegetables only cost 2·3 times as much as calories from the staple cereal, whereas calories from other vegetables cost 11·6 times as much, again more than in any other world region^(32)^. This is not true for other fruits though, their calories only cost 3·1 times as much as calories from the staple cereal, quite comparable to other world regions. Several experts mentioned in the meetings that seasonality is an issue for other vegetables, which could also partly explain the high prices. Another way to make vegetables more available in rural areas would be the implementation of private vegetable gardens together with appropriate information provision. Since other fruits are both available and affordable, and there is no evidence for food taboos related to them, they could be a potential solution for improving MDD. Several experts mentioned that they are not seen as suitable food for children, again something that could be addressed in a social and behavioural change communication campaign focusing on their nutritional value.

Despite their overall good availability and affordability, vitamin A-rich fruits and vegetables also separate children meeting and not meeting MDD. In detail, 66·3 per cent of children who do not achieve MDD also do not consume vitamin A-rich fruits and vegetables, whereas this number is only 15·1 per cent for children who achieve MDD. Vitamin A-rich fruits and vegetables, given their good availability and affordability, offer great potential for closing the MDD poverty gap. For children who consume four food groups, the difference in vitamin A-rich fruit and vegetable consumption to children who achieve MDD is much smaller though. Vitamin A-rich fruits and vegetables can therefore help to close the MDD poverty gap but will not immediately affect MDD poverty rates.

The prices of calories of fish and meat are comparable to other world regions. Particularly for the many coastal countries of the region, availability and affordability of fish is quite good^(33,34)^. However, our nationally representative data show that flesh foods, which include fish, separate children who meet the MDD from those who do not. Among children who do not meet the MDD, 67·9 per cent do not consume flesh foods, while this number drops to 12·6 per cent among children who meet the MDD. Whereas this difference is large overall, it is relatively small for children who already consume four food groups, thus similarly to vitamin A-rich fruits and vegetables, this food group can help to close the MDD poverty gap but will not immediately affect MDD poverty rates.

One of the strengths of this study is the use of nationally representative data from the DHS and MICS databases, which provide a comprehensive and mostly up-to-date overview of the dietary patterns of children aged 6–23 months for most countries in West and Central Africa. The detailed analysis, including the quantification of the MDD poverty rate and the MDD poverty gap, provides clear indicators to assess challenges and track changes in dietary diversity. By identifying specific food groups, such as eggs, legumes and nuts, that have the greatest potential to improve MDD levels, the study provides actionable insights for nutrition interventions. Through the inclusion of expert interviews, barriers to affordability and utilisation of these food groups will be identified: The contextualised recommendations, such as increasing the affordability of eggs through domestic production and addressing cultural taboos, are practical and tailored to the specific needs of the region.

Limitations of the study include that dietary data may be susceptible to reporting bias, as respondents may under- or over-report certain foods due to memory problems or socially desirable response behaviour. Despite regional findings, the study may not have fully captured cultural and regional variability within West and Central Africa, which could affect the generalisability of the results. Despite these limitations, this is a comprehensive descriptive study using additionally qualitative interviews that provides valuable new insights into the dietary diversity of children in West and Central Africa.

### Conclusion

We have quantified the MDD poverty rate as well as the MDD poverty gap for West and Central Africa. In detail, 82·0 percent of children between 6 and 23 months do not achieve MDD and on average are lacking 2·5 food groups. The MDD poverty gap is 40·4 per cent. The share of children who are lacking one food group to achieving MDD is larger than the share of children who already achieve MDD. Therefore, increasing consumption by only one food group could already make a big difference for the levels of MDD and increasing it by two food groups would make an even larger difference. In an analysis of individual food groups, we have identified those food groups which separate children meeting and not meeting MDD. Our findings can therefore inform the design of policy interventions to specifically target the provision of food groups which prevent children from achieving MDD. The qualitative insights about why some food groups are not consumed could help policymakers to design and evaluate interventions which specifically address barriers to consumption of important food groups.

## Supporting information

Vollmer et al. supplementary material Vollmer et al. supplementary material

## Supplementary material

For supplementary material accompanying this paper visit https://doi.org/10.1017/S1368980025000023
