# Supplementary material for: Measuring child food poverty: understanding the gap to achieving minimum dietary diversity
Source: Public Health Nutr. 2025 Jan 8;28(1):e27. doi: 10.1017/S1368980025000023 (PMC11822586; doi:10.1017/S1368980025000023)
Supplement: Vollmer et al. supplementary material [file S1368980025000023sup001.docx]

# Appendix:

# Discussion Questions in Focus Group Discussions:

# Accessibility:

# What are the specific foods from those food groups which can be a solution for children (availability criteria and no taboos) by country or ecological zone?

# What is the affordability of those selected foods by country of ecological zone?

# What could be done to increase access and affordability of those foods by ecological zone (from supply chains, environment, consumer behavior aspects)?

# Barriers

# What are the barriers for the specific foods access and affordability at community level and what should be taken into consideration to increase access and use?

# What are policies gaps for complementary feeding and national monitoring system?

# Results by ecological zone and by country

# Sahel: Mali, Burkina Faso, Niger, Mauritania, Chad

***Table 4:*** *Number of Food Groups Consumed by Children (6-23 months)*

|  | Observations | Percent | Cum |
| --- | --- | --- | --- |
| 0 | 257 | 1.7 | 1.7 |
| 1 | 2,870 | 19.4 | 21.1 |
| 2 | 3,613 | 24.4 | 45.5 |
| 3 | 2,952 | 19.9 | 65.4 |
| 4 | 2,376 | 16.0 | 81.5 |
| 5 | 1,437 | 9.7 | 91.2 |
| 6 | 716 | 4.8 | 96.0 |
| 7 | 424 | 2.9 | 98.9 |
| 8 | 166 | 1.1 | 100.0 |
| Total | 14,811 | 100.0 |  |

***Table 5****: Missing food groups for Children by MDD status (6-23 months)*

|  | MDD | No MDD | 4 FG | 3 FG |
| --- | --- | --- | --- | --- |
|  | Percent | Percent | Percent | Percent |
| Eggs | 54.5 | 95.9 | 88.6 | 94.5 |
| Legumes and Nuts | 51.1 | 91.7 | 78.6 | 88.4 |
| Other Fruits/ Vegetables | 47.6 | 93.7 | 82.4 | 91.3 |
| Dairy | 21.7 | 70.2 | 47.2 | 59.6 |
| Flesh Foods | 11.9 | 76.8 | 39.3 | 66.3 |
| Vitamin A Fruits/ Vegetables | 15.2 | 73.1 | 33.0 | 58.1 |
| Breastmilk | 17.4 | 17.3 | 16.7 | 17.8 |
| Grains, Roots, Tubers | 5.5 | 45.4 | 14.2 | 23.9 |
| Observations | 2,743 | 12,068 | 2,376 | 2,952 |

MDD: Minimum Dietary Diversity, FG: Food Groups

# Coast 1: Senegal, Gambia, Guinea Bissau, Guinea

***Table 6****: Number of Food Groups Consumed by Children (6-23 months)*

|  | Observations | Percent | Cum |
| --- | --- | --- | --- |
| 0 | 108 | 1.3 | 1.3 |
| 1 | 1,400 | 17.2 | 18.5 |
| 2 | 2,226 | 27.3 | 45.8 |
| 3 | 1,869 | 22.9 | 68.7 |
| 4 | 1,414 | 17.3 | 86.1 |
| 5 | 726 | 8.9 | 95.0 |
| 6 | 316 | 3.9 | 98.8 |
| 7 | 81 | 1.0 | 99.8 |
| 8 | 13 | 0.2 | 100.0 |
| Total | 8,153 | 100.0 |  |

***Table 7:*** *Missing food groups for Children by MDD status (6-23 months)*

|  | MDD | No MDD | 4 FG | 3 FG |
| --- | --- | --- | --- | --- |
|  | Percent | Percent | Percent | Percent |
| Eggs | 59.2 | 95.6 | 88.5 | 94.5 |
| Legumes and Nuts | 64.3 | 94.2 | 82.7 | 92.7 |
| Other Fruits/ Vegetables | 54.7 | 93.6 | 81.1 | 92.2 |
| Dairy | 30.8 | 80.7 | 60.6 | 72.1 |
| Flesh Foods | 9.2 | 70.7 | 24.8 | 57.2 |
| Vitamin A Fruits/ Vegetables | 18.7 | 78.2 | 42.4 | 68.4 |
| Breastmilk | 16.1 | 17.4 | 17.8 | 17.7 |
| Grains, Roots, Tubers | 1.4 | 25.8 | 2.1 | 5.2 |
| Observations | 1,136 | 7,017 | 1,414 | 1,869 |

MDD: Minimum Dietary Diversity, FG: Food Groups

## Coast 2: Ivory Coast, Benin, Ghana, Togo, Liberia, Sierra Leone

***Table 8****: Number of Food Groups Consumed by Children (6-23 months)*

|  | Observations | Percent | Cum |
| --- | --- | --- | --- |
| 0 | 130 | 0.9 | 0.9 |
| 1 | 2,345 | 15.9 | 16.8 |
| 2 | 3,024 | 20.6 | 37.4 |
| 3 | 3,314 | 22.5 | 59.9 |
| 4 | 2,795 | 19.0 | 78.9 |
| 5 | 1,680 | 11.4 | 90.3 |
| 6 | 842 | 5.7 | 96.1 |
| 7 | 401 | 2.7 | 98.8 |
| 8 | 179 | 1.2 | 100.0 |
| Total | 14,710 | 100.0 |  |

***Table 9:*** *Missing food groups for Children by MDD status (6-23 months)*

|  | MDD | No MDD | 4 FG | 3 FG |
| --- | --- | --- | --- | --- |
|  | Percent | Percent | Percent | Percent |
| Eggs | 51.1 | 93.6 | 84.7 | 92.8 |
| Legumes and Nuts | 47.0 | 91.6 | 80.6 | 90.2 |
| Other Fruits/ Vegetables | 36.5 | 90.1 | 75.2 | 89.4 |
| Dairy | 44.4 | 84.6 | 75.2 | 78.8 |
| Flesh Foods | 10.6 | 60.6 | 21.9 | 46.3 |
| Vitamin A Fruits/ Vegetables | 16.6 | 73.2 | 36.3 | 67.2 |
| Breastmilk | 19.1 | 20.9 | 19.5 | 24.1 |
| Grains, Roots, Tubers | 4.4 | 31.0 | 6.5 | 11.3 |
| Observations | 3,102 | 11,608 | 2,795 | 3,314 |

MDD: Minimum Dietary Diversity, FG: Food Groups

## Coast 3: Nigeria, Cameroon, Gabon, Sao Tome

***Table 10:*** *Number of Food Groups Consumed by Children (6-23 months)*

|  | Observations | Percent | Cum |
| --- | --- | --- | --- |
| 0 | 110 | 0.9 | 0.9 |
| 1 | 1,487 | 11.6 | 12.5 |
| 2 | 2,605 | 20.3 | 32.8 |
| 3 | 3,236 | 25.3 | 58.1 |
| 4 | 2,702 | 21.1 | 79.2 |
| 5 | 1,515 | 11.8 | 91.0 |
| 6 | 697 | 5.4 | 96.5 |
| 7 | 372 | 2.9 | 99.4 |
| 8 | 79 | 0.6 | 100.0 |
| Total | 12,803 | 100.0 |  |

***Table 11:*** *Missing food groups for Children by MDD status (6-23 months)*

|  | MDD | No MDD | 4 FG | 3 FG |
| --- | --- | --- | --- | --- |
|  | Percent | Percent | Percent | Percent |
| Eggs | 52.0 | 92.9 | 84.6 | 92.8 |
| Legumes and Nuts | 37.4 | 83.5 | 65.8 | 81.4 |
| Other Fruits/ Vegetables | 42.2 | 89.4 | 73.8 | 90.7 |
| Dairy | 36.5 | 77.9 | 65.6 | 73.0 |
| Flesh Foods | 18.3 | 69.1 | 41.6 | 62.6 |
| Vitamin A Fruits/ Vegetables | 17.2 | 65.7 | 33.6 | 58.8 |
| Breastmilk | 30.9 | 32.9 | 31.1 | 34.2 |
| Grains, Roots, Tubers | 2.4 | 20.4 | 3.8 | 6.4 |
| Observations | 2,663 | 10,140 | 2,702 | 3,236 |

MDD: Minimum Dietary Diversity, FG: Food Groups

## Central Africa: Central African Republic, Congo Democratic Republic, Congo

***Table 12:*** *Number of Food Groups Consumed by Children (6-23 months)*

|  | Observations | Percent | Cum |
| --- | --- | --- | --- |
| 0 | 171 | 1.5 | 1.5 |
| 1 | 1,412 | 12.0 | 13.4 |
| 2 | 2,682 | 22.8 | 36.2 |
| 3 | 3,404 | 28.9 | 65.1 |
| 4 | 2,538 | 21.5 | 86.6 |
| 5 | 1,097 | 9.3 | 96.0 |
| 6 | 352 | 3.0 | 98.9 |
| 7 | 105 | 0.9 | 99.8 |
| 8 | 19 | 0.2 | 100.0 |
| Total | 11,780 | 100.0 |  |

***Table 13:*** *Missing food groups for Children by MDD status (6-23 months)*

|  | MDD | No MDD | 4 FG | 3 FG |
| --- | --- | --- | --- | --- |
|  | Percent | Percent | Percent | Percent |
| Eggs | 71.3 | 96.6 | 91.8 | 96.8 |
| Legumes and Nuts | 57.7 | 91.3 | 80.8 | 91.5 |
| Other Fruits/ Vegetables | 37.6 | 89.4 | 74.3 | 90.5 |
| Dairy | 57.0 | 86.4 | 77.8 | 85.6 |
| Flesh Foods | 10.6 | 62.3 | 30.0 | 57.9 |
| Vitamin A Fruits/ Vegetables | 5.5 | 42.8 | 14.2 | 29.4 |
| Breastmilk | 15.9 | 26.5 | 18.8 | 24.8 |
| Grains, Roots, Tubers | 5.1 | 38.9 | 12.2 | 23.6 |
| Observations | 1,573 | 10,207 | 2,538 | 3,404 |

MDD: Minimum Dietary Diversity, FG: Food Groups

## Benin

***Table 14:*** *Number of Food Groups Consumed by Children (6-23 months)*

|  | Observations | Percent | Cum |
| --- | --- | --- | --- |
| 0 | 82 | 2.1 | 2.1 |
| 1 | 909 | 23.5 | 25.6 |
| 2 | 768 | 19.9 | 45.5 |
| 3 | 652 | 16.9 | 62.3 |
| 4 | 501 | 13.0 | 75.3 |
| 5 | 374 | 9.7 | 85.0 |
| 6 | 287 | 7.4 | 92.4 |
| 7 | 187 | 4.8 | 97.2 |
| 8 | 108 | 2.8 | 100.0 |
| Total | 3,868 | 100.0 |  |

***Table 15:*** *Missing food groups for Children by MDD status (6-23 months)*

|  | MDD | No MDD | 4 FG | 3 FG |
| --- | --- | --- | --- | --- |
|  | Percent | Percent | Percent | Percent |
| Eggs | 38.3 | 91.6 | 77.2 | 85.7 |
| Legumes and Nuts | 35.1 | 88.1 | 68.7 | 80.2 |
| Other Fruits/ Vegetables | 30.2 | 87.8 | 64.3 | 82.1 |
| Dairy | 28.1 | 82.9 | 59.9 | 73.9 |
| Flesh Foods | 8.7 | 67.7 | 30.1 | 44.6 |
| Vitamin A Fruits/ Vegetables | 24.9 | 84.1 | 55.7 | 74.4 |
| Breastmilk | 20.4 | 20.2 | 19.8 | 24.8 |
| Grains, Roots, Tubers | 11.2 | 57.7 | 24.4 | 34.2 |
| Observations | 956 | 2,912 | 501 | 652 |

MDD: Minimum Dietary Diversity, FG: Food Groups

## Burkina Faso

***Table 16:*** *Number of Food Groups Consumed by Children (6-23 months)*

|  | Observations | Percent | Cum |
| --- | --- | --- | --- |
| 0 | 12 | 0.6 | 0.6 |
| 1 | 517 | 25.0 | 25.6 |
| 2 | 803 | 38.9 | 64.5 |
| 3 | 413 | 20.0 | 84.5 |
| 4 | 225 | 10.9 | 95.4 |
| 5 | 71 | 3.4 | 98.8 |
| 6 | 13 | 0.6 | 99.5 |
| 7 | 9 | 0.4 | 99.9 |
| 8 | 2 | 0.1 | 100.0 |
| Total | 2,065 | 100.0 |  |

***Table 17:*** *Missing food groups for Children by MDD status(6-23 months)*

|  | MDD | No MDD | 4 FG | 3 FG |
| --- | --- | --- | --- | --- |
|  | Percent | Percent | Percent | Percent |
| Eggs | 53.7 | 96.9 | 84.4 | 94.9 |
| Legumes and Nuts | 53.7 | 95.1 | 81.8 | 90.6 |
| Other Fruits/ Vegetables | 54.7 | 96.5 | 84.0 | 92.7 |
| Dairy | 45.3 | 90.6 | 76.0 | 82.3 |
| Flesh Foods | 15.8 | 82.4 | 29.8 | 62.5 |
| Vitamin A Fruits/ Vegetables | 26.3 | 80.1 | 31.1 | 56.9 |
| Breastmilk | 7.4 | 6.3 | 7.1 | 7.0 |
| Grains, Roots, Tubers | 4.2 | 35.7 | 5.8 | 13.1 |
| Observations | 95 | 1,970 | 225 | 413 |

MDD: Minimum Dietary Diversity, FG: Food Groups

## Cameroon

***Table 18****: Number of Food Groups Consumed by Children (6-23 months)*

|  | Observations | Percent | Cum |
| --- | --- | --- | --- |
| 0 | 37 | 1.5 | 1.5 |
| 1 | 255 | 10.0 | 11.5 |
| 2 | 475 | 18.6 | 30.1 |
| 3 | 726 | 28.5 | 58.6 |
| 4 | 611 | 24.0 | 82.5 |
| 5 | 285 | 11.2 | 93.7 |
| 6 | 101 | 4.0 | 97.7 |
| 7 | 50 | 2.0 | 99.6 |
| 8 | 9 | 0.4 | 100.0 |
| Total | 2,549 | 100.0 |  |

***Table 19:*** *Missing food groups for Children by MDD status (6-23 months)*

|  | MDD | No MDD | 4 FG | 3 FG |
| --- | --- | --- | --- | --- |
|  | Percent | Percent | Percent | Percent |
| Eggs | 55.1 | 92.0 | 85.9 | 90.6 |
| Legumes and Nuts | 63.4 | 93.8 | 88.7 | 92.6 |
| Other Fruits/ Vegetables | 18.0 | 76.2 | 49.6 | 78.4 |
| Dairy | 52.1 | 84.1 | 74.8 | 82.4 |
| Flesh Foods | 8.8 | 55.8 | 26.4 | 50.4 |
| Vitamin A Fruits/ Vegetables | 13.5 | 58.9 | 35.0 | 51.7 |
| Breastmilk | 35.5 | 43.4 | 35.5 | 46.4 |
| Grains, Roots, Tubers | 2.5 | 18.7 | 4.1 | 7.6 |
| Observations | 445 | 2,104 | 611 | 726 |

MDD: Minimum Dietary Diversity, FG: Food Groups

## Central African Republic

23

***Table 20:*** *Number of Food Groups Consumed by Children (6-23 months)*

|  | Observations | Percent | Cum |
| --- | --- | --- | --- |
| 0 | 89 | 3.5 | 3.5 |
| 1 | 431 | 17.1 | 20.7 |
| 2 | 683 | 27.1 | 47.8 |
| 3 | 661 | 26.3 | 74.1 |
| 4 | 395 | 15.7 | 89.8 |
| 5 | 158 | 6.3 | 96.1 |
| 6 | 70 | 2.8 | 98.8 |
| 7 | 24 | 1.0 | 99.8 |
| 8 | 5 | 0.2 | 100.0 |
| Total | 2,516 | 100.0 |  |

***Table 21****: Missing food groups for Children by MDD status (6-23 months)*

|  | MDD | No MDD | 4 FG | 3 FG |
| --- | --- | --- | --- | --- |
|  | Percent | Percent | Percent | Percent |
| Eggs | 67.7 | 97.9 | 91.4 | 98.3 |
| Legumes and Nuts | 41.6 | 86.6 | 62.3 | 83.7 |
| Other Fruits/ Vegetables | 40.1 | 93.8 | 80.3 | 93.2 |
| Dairy | 50.6 | 92.6 | 80.5 | 91.8 |
| Flesh Foods | 20.2 | 79.7 | 46.8 | 76.7 |
| Vitamin A Fruits/ Vegetables | 5.8 | 43.6 | 10.6 | 19.4 |
| Breastmilk | 16.7 | 24.4 | 17.2 | 18.6 |
| Grains, Roots, Tubers | 5.4 | 44.1 | 10.9 | 18.3 |
| Observations | 257 | 2,259 | 395 | 661 |

MDD: Minimum Dietary Diversity, FG: Food Groups

## Chad

24

***Table 22:*** *Number of Food Groups Consumed by Children (6-23 months)*

|  | Observations | Percent | Cum |
| --- | --- | --- | --- |
| 0 | 125 | 2.3 | 2.3 |
| 1 | 954 | 17.6 | 19.9 |
| 2 | 1,136 | 21.0 | 40.9 |
| 3 | 1,005 | 18.5 | 59.4 |
| 4 | 918 | 16.9 | 76.3 |
| 5 | 609 | 11.2 | 87.6 |
| 6 | 359 | 6.6 | 94.2 |
| 7 | 227 | 4.2 | 98.4 |
| 8 | 89 | 1.6 | 100.0 |
| Total | 5,422 | 100.0 |  |

***Table 23****: Missing food groups for Children by MDD status (6-23 months)*

|  | MDD | No MDD | 4 FG | 3 FG |
| --- | --- | --- | --- | --- |
|  | Percent | Percent | Percent | Percent |
| Eggs | 53.0 | 93.9 | 85.6 | 92.0 |
| Legumes and Nuts | 43.8 | 89.9 | 76.4 | 86.1 |
| Other Fruits/ Vegetables | 42.8 | 89.1 | 73.9 | 84.8 |
| Dairy | 22.9 | 67.2 | 48.0 | 59.6 |
| Flesh Foods | 10.9 | 72.2 | 35.8 | 61.4 |
| Vitamin A Fruits/ Vegetables | 16.0 | 70.6 | 37.4 | 56.8 |
| Breastmilk | 18.2 | 19.9 | 18.2 | 19.2 |
| Grains, Roots, Tubers | 8.3 | 57.6 | 24.7 | 40.1 |
| Observations | 1,284 | 4,138 | 918 | 1,005 |

MDD: Minimum Dietary Diversity, FG: Food Groups

## Congo

25

***Table 24:*** *Number of Food Groups Consumed by Children (6-23 months)*

|  | Observations | Percent | Cum |
| --- | --- | --- | --- |
| 0 | 33 | 1.2 | 1.2 |
| 1 | 300 | 10.8 | 12.0 |
| 2 | 707 | 25.6 | 37.6 |
| 3 | 801 | 29.0 | 66.6 |
| 4 | 580 | 21.0 | 87.6 |
| 5 | 243 | 8.8 | 96.3 |
| 6 | 73 | 2.6 | 99.0 |
| 7 | 24 | 0.9 | 99.9 |
| 8 | 4 | 0.1 | 100.0 |
| Total | 2,765 | 100.0 |  |

***Table 25:*** *Missing food groups for Children by MDD status (6-23 months)*

|  | MDD | No MDD | 4 FG | 3 FG |
| --- | --- | --- | --- | --- |
|  | Percent | Percent | Percent | Percent |
| Eggs | 75.6 | 98.0 | 95.2 | 98.0 |
| Legumes and Nuts | 52.9 | 94.4 | 86.2 | 94.6 |
| Other Fruits/ Vegetables | 58.1 | 95.4 | 88.6 | 96.3 |
| Dairy | 27.0 | 62.7 | 44.7 | 58.7 |
| Flesh Foods | 7.6 | 54.6 | 20.7 | 43.9 |
| Vitamin A Fruits/ Vegetables | 9.3 | 63.6 | 28.8 | 58.1 |
| Breastmilk | 28.5 | 40.4 | 33.3 | 39.7 |
| Grains, Roots, Tubers | 2.3 | 25.1 | 2.6 | 10.7 |
| Observations | 344 | 2,421 | 580 | 801 |

MDD: Minimum Dietary Diversity, FG: Food Groups

## Democratic Republic of Congo

26

***Table 26****: Number of Food Groups Consumed by Children (6-23 months)*

|  | Observations | Percent | Cum |
| --- | --- | --- | --- |
| 0 | 49 | 0.8 | 0.8 |
| 1 | 681 | 10.5 | 11.2 |
| 2 | 1,292 | 19.9 | 31.1 |
| 3 | 1,942 | 29.9 | 61.0 |
| 4 | 1,563 | 24.0 | 85.0 |
| 5 | 696 | 10.7 | 95.8 |
| 6 | 209 | 3.2 | 99.0 |
| 7 | 57 | 0.9 | 99.8 |
| 8 | 10 | 0.2 | 100.0 |
| Total | 6,499 | 100.0 |  |

***Table 27:*** *Missing food groups for Children by MDD status (6-23 months)*

|  | MDD | No MDD | 4 FG | 3 FG |
| --- | --- | --- | --- | --- |
|  | Percent | Percent | Percent | Percent |
| Eggs | 70.8 | 95.4 | 90.7 | 95.8 |
| Legumes and Nuts | 63.7 | 91.8 | 83.5 | 92.8 |
| Other Fruits/ Vegetables | 29.6 | 84.9 | 67.5 | 87.2 |
| Dairy | 69.2 | 94.3 | 89.4 | 94.5 |
| Flesh Foods | 9.1 | 58.6 | 29.2 | 57.3 |
| Vitamin A Fruits/ Vegetables | 4.0 | 33.4 | 9.7 | 21.0 |
| Breastmilk | 11.2 | 21.2 | 13.8 | 20.7 |
| Grains, Roots, Tubers | 6.1 | 42.8 | 16.1 | 30.7 |
| Observations | 972 | 5,527 | 1,563 | 1,942 |

MDD: Minimum Dietary Diversity, FG: Food Groups

## Gabon

27

***Table 28:*** *Number of Food Groups Consumed by Children (6-23 months)*

|  | Observations | Percent | Cum |
| --- | --- | --- | --- |
| 0 | 18 | 1.5 | 1.5 |
| 1 | 126 | 10.7 | 12.2 |
| 2 | 306 | 26.0 | 38.3 |
| 3 | 342 | 29.1 | 67.3 |
| 4 | 228 | 19.4 | 86.7 |
| 5 | 108 | 9.2 | 95.9 |
| 6 | 33 | 2.8 | 98.7 |
| 7 | 13 | 1.1 | 99.8 |
| 8 | 2 | 0.2 | 100.0 |
| Total | 1,176 | 100.0 |  |

***Table 29****: Missing food groups for Children by MDD status (6-23 months)*

|  | MDD | No MDD | 4 FG | 3 FG |
| --- | --- | --- | --- | --- |
|  | Percent | Percent | Percent | Percent |
| Eggs | 47.4 | 94.9 | 86.0 | 96.2 |
| Legumes and Nuts | 82.7 | 98.2 | 93.4 | 99.4 |
| Other Fruits/ Vegetables | 50.0 | 91.7 | 78.9 | 92.7 |
| Dairy | 7.1 | 50.7 | 26.3 | 42.4 |
| Flesh Foods | 11.5 | 56.8 | 21.9 | 45.3 |
| Vitamin A Fruits/ Vegetables | 12.8 | 68.1 | 36.8 | 66.1 |
| Breastmilk | 46.2 | 52.8 | 51.8 | 48.0 |
| Grains, Roots, Tubers | 0.6 | 24.4 | 4.8 | 9.9 |
| Observations | 156 | 1,020 | 228 | 342 |

MDD: Minimum Dietary Diversity, FG: Food Groups

## Gambia

28

***Table 30:*** *Number of Food Groups Consumed by Children (6-23 months)*

|  | Observations | Percent | Cum |
| --- | --- | --- | --- |
| 0 | 9 | 0.4 | 0.4 |
| 1 | 216 | 9.4 | 9.8 |
| 2 | 590 | 25.6 | 35.4 |
| 3 | 572 | 24.8 | 60.3 |
| 4 | 491 | 21.3 | 81.6 |
| 5 | 265 | 11.5 | 93.1 |
| 6 | 124 | 5.4 | 98.5 |
| 7 | 31 | 1.3 | 99.8 |
| 8 | 4 | 0.2 | 100.0 |
| Total | 2,302 | 100.0 |  |

***Table 31:*** *Missing food groups for Children by MDD status (6-23 months)*

|  | MDD | No MDD | 4 FG | 3 FG |
| --- | --- | --- | --- | --- |
|  | Percent | Percent | Percent | Percent |
| Eggs | 64.9 | 94.9 | 89.6 | 93.7 |
| Legumes and Nuts | 53.3 | 88.0 | 70.9 | 87.2 |
| Other Fruits/ Vegetables | 45.0 | 91.6 | 79.2 | 91.6 |
| Dairy | 31.6 | 76.0 | 54.2 | 69.2 |
| Flesh Foods | 8.7 | 60.6 | 19.6 | 47.4 |
| Vitamin A Fruits/ Vegetables | 34.2 | 86.9 | 66.8 | 86.7 |
| Breastmilk | 14.4 | 16.7 | 17.7 | 20.8 |
| Grains, Roots, Tubers | 1.2 | 14.9 | 2.0 | 3.3 |
| Observations | 424 | 1,878 | 491 | 572 |

MDD: Minimum Dietary Diversity, FG: Food Groups

## Ghana

***Table 32:*** *Number of Food Groups Consumed by Children (6-23 months)*

|  | Observations | Percent | Cum |
| --- | --- | --- | --- |
| 0 | 9 | 0.3 | 0.3 |
| 1 | 292 | 11.3 | 11.7 |
| 2 | 589 | 22.9 | 34.6 |
| 3 | 623 | 24.2 | 58.8 |
| 4 | 479 | 18.6 | 77.4 |
| 5 | 363 | 14.1 | 91.5 |
| 6 | 166 | 6.4 | 97.9 |
| 7 | 43 | 1.7 | 99.6 |
| 8 | 11 | 0.4 | 100.0 |
| Total | 2,575 | 100.0 |  |

***Table 33:*** *Missing food groups for Children by MDD status (6-23 months)*

|  | MDD | No MDD | 4 FG | 3 FG |
| --- | --- | --- | --- | --- |
|  | Percent | Percent | Percent | Percent |
| Eggs | 51.5 | 90.7 | 78.7 | 89.4 |
| Legumes and Nuts | 56.3 | 90.5 | 76.2 | 90.5 |
| Other Fruits/ Vegetables | 29.5 | 83.4 | 61.6 | 80.9 |
| Dairy | 56.1 | 86.9 | 76.8 | 81.7 |
| Flesh Foods | 17.5 | 69.2 | 34.0 | 63.4 |
| Vitamin A Fruits/ Vegetables | 18.9 | 74.0 | 45.1 | 65.5 |
| Breastmilk | 19.2 | 21.6 | 24.8 | 22.8 |
| Grains, Roots, Tubers | 2.2 | 19.8 | 2.7 | 5.8 |
| Observations | 583 | 1,992 | 479 | 623 |

MDD: Minimum Dietary Diversity, FG: Food Groups

## Guinea

***Table 34:*** *Number of Food Groups Consumed by Children (6-23 months)*

|  | Observations | Percent | Cum |
| --- | --- | --- | --- |
| 0 | 68 | 3.6 | 3.6 |
| 1 | 436 | 23.0 | 26.6 |
| 2 | 523 | 27.6 | 54.1 |
| 3 | 376 | 19.8 | 73.9 |
| 4 | 243 | 12.8 | 86.7 |
| 5 | 132 | 7.0 | 93.7 |
| 6 | 86 | 4.5 | 98.2 |
| 7 | 27 | 1.4 | 99.6 |
| 8 | 7 | 0.4 | 100.0 |
| Total | 1,898 | 100.0 |  |

***Table 35****: Missing food groups for Children by MDD status (6-23 months)*

|  | MDD | No MDD | 4 FG | 3 FG |
| --- | --- | --- | --- | --- |
|  | Percent | Percent | Percent | Percent |
| Eggs | 24.6 | 91.1 | 70.4 | 88.3 |
| Legumes and Nuts | 81.0 | 99.6 | 97.9 | 99.7 |
| Other Fruits/ Vegetables | 55.6 | 96.0 | 83.1 | 94.4 |
| Dairy | 30.6 | 81.3 | 60.1 | 67.3 |
| Flesh Foods | 14.3 | 86.1 | 40.7 | 83.0 |
| Vitamin A Fruits/ Vegetables | 7.5 | 73.0 | 27.2 | 46.5 |
| Breastmilk | 19.8 | 17.2 | 15.6 | 10.9 |
| Grains, Roots, Tubers | 2.8 | 38.1 | 4.9 | 9.8 |
| Observations | 252 | 1,646 | 243 | 376 |

MDD: Minimum Dietary Diversity, FG: Food Groups

## Guinea-Bissau

***Table 36:*** *Number of Food Groups Consumed by Children (6-23 months)*

|  | Observations | Percent | Cum |
| --- | --- | --- | --- |
| 0 | 26 | 1.2 | 1.2 |
| 1 | 500 | 22.8 | 24.0 |
| 2 | 692 | 31.6 | 55.5 |
| 3 | 518 | 23.6 | 79.2 |
| 4 | 311 | 14.2 | 93.3 |
| 5 | 106 | 4.8 | 98.2 |
| 6 | 29 | 1.3 | 99.5 |
| 7 | 10 | 0.5 | 100.0 |
| 8 | 1 | 0.0 | 100.0 |
| Total | 2,193 | 100.0 |  |

***Table 37:*** *Missing food groups for Children by MDD status (6-23 months)*

|  | MDD | No MDD | 4 FG | 3 FG |
| --- | --- | --- | --- | --- |
|  | Percent | Percent | Percent | Percent |
| Eggs | 75.3 | 98.1 | 92.0 | 98.3 |
| Legumes and Nuts | 52.7 | 93.9 | 80.1 | 90.9 |
| Other Fruits/ Vegetables | 37.7 | 91.6 | 69.8 | 89.0 |
| Dairy | 51.4 | 89.4 | 72.0 | 84.6 |
| Flesh Foods | 11.6 | 68.9 | 21.9 | 43.8 |
| Vitamin A Fruits/ Vegetables | 18.5 | 83.7 | 43.1 | 74.3 |
| Breastmilk | 15.8 | 17.0 | 19.6 | 15.1 |
| Grains, Roots, Tubers | 1.4 | 28.4 | 1.6 | 4.1 |
| Observations | 146 | 2,047 | 311 | 518 |

MDD: Minimum Dietary Diversity, FG: Food Groups

## Ivory Coast

***Table 38****: Number of Food Groups Consumed by Children (6-23 months)*

|  | Observations | Percent | Cum |
| --- | --- | --- | --- |
| 0 | 8 | 0.3 | 0.3 |
| 1 | 367 | 13.8 | 14.1 |
| 2 | 529 | 19.8 | 33.9 |
| 3 | 704 | 26.4 | 60.3 |
| 4 | 593 | 22.2 | 82.5 |
| 5 | 309 | 11.6 | 94.1 |
| 6 | 117 | 4.4 | 98.5 |
| 7 | 40 | 1.5 | 100.0 |
| 8 | 1 | 0.0 | 100.0 |
| Total | 2,668 | 100.0 |  |

***Table 39:*** *Missing food groups for Children by MDD status (6-23 months)*

|  | MDD | No MDD | 4 FG | 3 FG |
| --- | --- | --- | --- | --- |
|  | Percent | Percent | Percent | Percent |
| Eggs | 65.3 | 94.5 | 86.7 | 95.3 |
| Legumes and Nuts | 62.7 | 93.5 | 83.0 | 94.5 |
| Other Fruits/ Vegetables | 41.5 | 91.1 | 75.0 | 93.9 |
| Dairy | 51.0 | 82.5 | 71.0 | 80.0 |
| Flesh Foods | 5.4 | 50.7 | 17.9 | 34.1 |
| Vitamin A Fruits/ Vegetables | 14.8 | 73.8 | 41.1 | 72.2 |
| Breastmilk | 15.6 | 24.1 | 23.8 | 25.4 |
| Grains, Roots, Tubers | 0.9 | 21.5 | 1.5 | 4.7 |
| Observations | 467 | 2,201 | 593 | 704 |

MDD: Minimum Dietary Diversity, FG: Food Groups

## Liberia

***Table 40:*** *Number of Food Groups Consumed by Children (6-23 months)*

|  | Observations | Percent | Cum |
| --- | --- | --- | --- |
| 0 | 9 | 0.6 | 0.6 |
| 1 | 311 | 20.6 | 21.2 |
| 2 | 294 | 19.5 | 40.7 |
| 3 | 430 | 28.5 | 69.3 |
| 4 | 333 | 22.1 | 91.4 |
| 5 | 88 | 5.8 | 97.2 |
| 6 | 22 | 1.5 | 98.7 |
| 7 | 16 | 1.1 | 99.7 |
| 8 | 4 | 0.3 | 100.0 |
| Total | 1,507 | 100.0 |  |

***Table 41****: Missing food groups for Children by MDD status (6-23 months)*

|  | MDD | No MDD | 4 FG | 3 FG |
| --- | --- | --- | --- | --- |
|  | Percent | Percent | Percent | Percent |
| Eggs | 56.9 | 97.5 | 94.3 | 97.2 |
| Legumes and Nuts | 66.2 | 95.8 | 90.4 | 95.1 |
| Other Fruits/ Vegetables | 26.9 | 94.2 | 82.6 | 96.0 |
| Dairy | 69.2 | 93.2 | 92.8 | 91.6 |
| Flesh Foods | 6.9 | 53.3 | 8.7 | 36.3 |
| Vitamin A Fruits/ Vegetables | 10.8 | 65.2 | 19.5 | 58.1 |
| Breastmilk | 12.3 | 18.2 | 10.5 | 21.6 |
| Grains, Roots, Tubers | 0.0 | 26.9 | 1.2 | 4.0 |
| Observations | 130 | 1,377 | 333 | 430 |

MDD: Minimum Dietary Diversity, FG: Food Groups

## Mali

***Table 42:*** *Number of Food Groups Consumed by Children (6-23 months)*

|  | Observations | Percent | Cum |
| --- | --- | --- | --- |
| 0 | 51 | 1.9 | 1.9 |
| 1 | 591 | 21.9 | 23.7 |
| 2 | 519 | 19.2 | 42.9 |
| 3 | 524 | 19.4 | 62.3 |
| 4 | 445 | 16.5 | 78.8 |
| 5 | 308 | 11.4 | 90.2 |
| 6 | 145 | 5.4 | 95.5 |
| 7 | 88 | 3.3 | 98.8 |
| 8 | 33 | 1.2 | 100.0 |
| Total | 2,704 | 100.0 |  |

***Table 43:*** *Missing food groups for Children by MDD status (6-23 months)*

|  | MDD | No MDD | 4 FG | 3 FG |
| --- | --- | --- | --- | --- |
|  | Percent | Percent | Percent | Percent |
| Eggs | 44.1 | 95.9 | 88.1 | 94.5 |
| Legumes and Nuts | 65.0 | 95.7 | 84.5 | 96.4 |
| Other Fruits/ Vegetables | 49.8 | 97.2 | 89.9 | 97.7 |
| Dairy | 28.0 | 78.5 | 63.8 | 71.4 |
| Flesh Foods | 7.3 | 67.7 | 22.5 | 51.5 |
| Vitamin A Fruits/ Vegetables | 15.0 | 73.9 | 31.9 | 61.1 |
| Breastmilk | 15.5 | 17.1 | 13.5 | 15.8 |
| Grains, Roots, Tubers | 2.1 | 40.2 | 5.8 | 11.6 |
| Observations | 574 | 2,130 | 445 | 524 |

MDD: Minimum Dietary Diversity, FG: Food Groups

## Mauritania

***Table 44:*** *Number of Food Groups Consumed by Children (6-23 months)*

|  | Observations | Percent | Cum |
| --- | --- | --- | --- |
| 0 | 51 | 1.7 | 1.7 |
| 1 | 470 | 15.4 | 17.0 |
| 2 | 629 | 20.5 | 37.6 |
| 3 | 681 | 22.2 | 59.8 |
| 4 | 616 | 20.1 | 79.9 |
| 5 | 351 | 11.5 | 91.4 |
| 6 | 155 | 5.1 | 96.5 |
| 7 | 74 | 2.4 | 98.9 |
| 8 | 34 | 1.1 | 100.0 |
| Total | 3,061 | 100.0 |  |

***Table 45:*** *Missing food groups for Children by MDD status (6-23 months)*

|  | MDD | No MDD | 4 FG | 3 FG |
| --- | --- | --- | --- | --- |
|  | Percent | Percent | Percent | Percent |
| Eggs | 67.9 | 97.7 | 94.0 | 97.8 |
| Legumes and Nuts | 55.2 | 89.8 | 81.7 | 86.9 |
| Other Fruits/ Vegetables | 53.3 | 94.0 | 88.1 | 93.1 |
| Dairy | 6.2 | 43.8 | 18.8 | 32.7 |
| Flesh Foods | 16.0 | 80.8 | 55.5 | 77.7 |
| Vitamin A Fruits/ Vegetables | 12.7 | 68.7 | 30.0 | 60.1 |
| Breastmilk | 18.9 | 23.9 | 20.8 | 27.3 |
| Grains, Roots, Tubers | 3.9 | 46.5 | 11.0 | 24.4 |
| Observations | 614 | 2,447 | 616 | 681 |

MDD: Minimum Dietary Diversity, FG: Food Groups

## Niger

36

***Table 46:*** *Number of Food Groups Consumed by Children (6-23 months)*

|  | Observations | Percent | Cum |
| --- | --- | --- | --- |
| 0 | 18 | 1.2 | 1.2 |
| 1 | 338 | 21.7 | 22.8 |
| 2 | 526 | 33.7 | 56.6 |
| 3 | 329 | 21.1 | 77.7 |
| 4 | 172 | 11.0 | 88.7 |
| 5 | 98 | 6.3 | 95.0 |
| 6 | 44 | 2.8 | 97.8 |
| 7 | 26 | 1.7 | 99.5 |
| 8 | 8 | 0.5 | 100.0 |
| Total | 1,559 | 100.0 |  |

***Table 47:*** *Missing food groups for Children by MDD status (6-23 months)*

|  | MDD | No MDD | 4 FG | 3 FG |
| --- | --- | --- | --- | --- |
|  | Percent | Percent | Percent | Percent |
| Eggs | 52.8 | 97.7 | 91.9 | 95.1 |
| Legumes and Nuts | 43.2 | 89.7 | 60.5 | 83.3 |
| Other Fruits/ Vegetables | 51.1 | 97.0 | 86.6 | 95.7 |
| Dairy | 33.0 | 84.3 | 64.0 | 68.1 |
| Flesh Foods | 18.2 | 90.0 | 55.8 | 85.7 |
| Vitamin A Fruits/ Vegetables | 13.6 | 77.4 | 25.0 | 55.0 |
| Breastmilk | 18.2 | 13.9 | 14.5 | 10.0 |
| Grains, Roots, Tubers | 1.7 | 28.3 | 1.7 | 7.0 |
| Observations | 176 | 1,383 | 172 | 329 |

MDD: Minimum Dietary Diversity, FG: Food Groups

## Nigeria

37

***Table 48:*** *Number of Food Groups Consumed by Children (6-23 months)*

|  | Observations | Percent | Cum |
| --- | --- | --- | --- |
| 0 | 49 | 0.6 | 0.6 |
| 1 | 1,062 | 12.4 | 13.0 |
| 2 | 1,762 | 20.5 | 33.5 |
| 3 | 2,056 | 24.0 | 57.5 |
| 4 | 1,736 | 20.2 | 77.7 |
| 5 | 1,023 | 11.9 | 89.7 |
| 6 | 526 | 6.1 | 95.8 |
| 7 | 295 | 3.4 | 99.2 |
| 8 | 66 | 0.8 | 100.0 |
| Total | 8,575 | 100.0 |  |

***Table 49:*** *Missing food groups for Children by MDD status (6-23 months)*

|  | MDD | No MDD | 4 FG | 3 FG |
| --- | --- | --- | --- | --- |
|  | Percent | Percent | Percent | Percent |
| Eggs | 50.7 | 92.9 | 84.0 | 92.9 |
| Legumes and Nuts | 25.0 | 77.4 | 52.6 | 73.6 |
| Other Fruits/ Vegetables | 48.2 | 93.5 | 82.3 | 94.8 |
| Dairy | 35.9 | 80.8 | 68.5 | 75.4 |
| Flesh Foods | 21.4 | 76.1 | 50.6 | 70.7 |
| Vitamin A Fruits/ Vegetables | 19.2 | 68.0 | 33.2 | 60.4 |
| Breastmilk | 28.5 | 26.0 | 25.6 | 27.4 |
| Grains, Roots, Tubers | 2.4 | 19.7 | 3.2 | 4.7 |
| Observations | 1,910 | 6,665 | 1,736 | 2,056 |

MDD: Minimum Dietary Diversity, FG: Food Groups

## Sao Tome and Principe

38

***Table 50****: Number of Food Groups Consumed by Children (6-23 months)*

|  | Observations | Percent | Cum |
| --- | --- | --- | --- |
| 0 | 6 | 1.2 | 1.2 |
| 1 | 44 | 8.7 | 9.9 |
| 2 | 62 | 12.3 | 22.3 |
| 3 | 112 | 22.3 | 44.5 |
| 4 | 127 | 25.2 | 69.8 |
| 5 | 99 | 19.7 | 89.5 |
| 6 | 37 | 7.4 | 96.8 |
| 7 | 14 | 2.8 | 99.6 |
| 8 | 2 | 0.4 | 100.0 |
| Total | 503 | 100.0 |  |

***Table 51****: Missing food groups for Children by MDD status (6-23 months)*

|  | MDD | No MDD | 4 FG | 3 FG |
| --- | --- | --- | --- | --- |
|  | Percent | Percent | Percent | Percent |
| Eggs | 63.8 | 91.2 | 83.5 | 92.9 |
| Legumes and Nuts | 69.7 | 94.0 | 86.6 | 97.3 |
| Other Fruits/ Vegetables | 30.3 | 83.2 | 65.4 | 89.3 |
| Dairy | 28.9 | 64.7 | 52.8 | 62.5 |
| Flesh Foods | 15.1 | 51.3 | 28.3 | 44.6 |
| Vitamin A Fruits/ Vegetables | 8.6 | 54.4 | 26.8 | 54.5 |
| Breastmilk | 31.6 | 42.7 | 47.2 | 38.4 |
| Grains, Roots, Tubers | 5.3 | 30.2 | 9.4 | 20.5 |
| Observations | 152 | 351 | 127 | 112 |

MDD: Minimum Dietary Diversity, FG: Food Groups

39

## Senegal

***Table 52:*** *Number of Food Groups Consumed by Children (6-23 months)*

|  | Observations | Percent | Cum |
| --- | --- | --- | --- |
| 0 | 5 | 0.3 | 0.3 |
| 1 | 248 | 14.1 | 14.4 |
| 2 | 421 | 23.9 | 38.3 |
| 3 | 403 | 22.9 | 61.2 |
| 4 | 369 | 21.0 | 82.2 |
| 5 | 223 | 12.7 | 94.8 |
| 6 | 77 | 4.4 | 99.2 |
| 7 | 13 | 0.7 | 99.9 |
| 8 | 1 | 0.1 | 100.0 |
| Total | 1,760 | 100.0 |  |

***Table 53:*** *Missing food groups for Children (6-23 months)*

|  | MDD | No MDD | 4 FG | 3 FG |
| --- | --- | --- | --- | --- |
|  | Percent | Percent | Percent | Percent |
| Eggs | 72.0 | 97.8 | 95.9 | 96.5 |
| Legumes and Nuts | 71.3 | 96.5 | 90.8 | 96.0 |
| Other Fruits/ Vegetables | 74.8 | 96.3 | 91.9 | 95.0 |
| Dairy | 20.4 | 73.9 | 59.9 | 64.5 |
| Flesh Foods | 4.8 | 68.7 | 23.6 | 64.5 |
| Vitamin A Fruits/ Vegetables | 6.7 | 64.8 | 19.5 | 55.1 |
| Breastmilk | 15.6 | 18.9 | 17.6 | 23.1 |
| Grains, Roots, Tubers | 0.6 | 22.1 | 0.8 | 5.2 |
| Observations | 314 | 1,446 | 369 | 403 |

MDD: Minimum Dietary Diversity, FG: Food Groups

## Sierra Leone

40

***Table 54:*** *Number of Food Groups Consumed by Children (6-23 months)*

|  | Observations | Percent | Cum |
| --- | --- | --- | --- |
| 0 | 21 | 0.8 | 0.8 |
| 1 | 308 | 11.7 | 12.5 |
| 2 | 557 | 21.2 | 33.7 |
| 3 | 561 | 21.3 | 55.0 |
| 4 | 482 | 18.3 | 73.3 |
| 5 | 342 | 13.0 | 86.3 |
| 6 | 199 | 7.6 | 93.8 |
| 7 | 107 | 4.1 | 97.9 |
| 8 | 55 | 2.1 | 100.0 |
| Total | 2,632 | 100.0 |  |

***Table 55****: Missing food groups for Children by MDD status (6-23 months)*

|  | MDD | No MDD | 4 FG | 3 FG |
| --- | --- | --- | --- | --- |
|  | Percent | Percent | Percent | Percent |
| Eggs | 50.9 | 95.0 | 83.6 | 96.8 |
| Legumes and Nuts | 43.8 | 92.8 | 83.0 | 91.6 |
| Other Fruits/ Vegetables | 32.7 | 92.7 | 80.7 | 92.5 |
| Dairy | 43.8 | 75.9 | 69.5 | 63.1 |
| Flesh Foods | 14.1 | 64.1 | 25.9 | 55.3 |
| Vitamin A Fruits/ Vegetables | 8.0 | 70.0 | 29.3 | 67.0 |
| Breastmilk | 22.9 | 23.8 | 22.2 | 26.0 |
| Grains, Roots, Tubers | 1.6 | 24.7 | 5.8 | 7.7 |
| Observations | 703 | 1,929 | 482 | 561 |

MDD: Minimum Dietary Diversity, FG: Food Groups

## Togo

***Table 56****: Number of Food Groups Consumed by Children (6-23 months)*

|  | Observations | Percent | Cum |
| --- | --- | --- | --- |
| 0 | 1 | 0.1 | 0.1 |
| 1 | 158 | 10.8 | 10.9 |
| 2 | 287 | 19.7 | 30.5 |
| 3 | 344 | 23.6 | 54.1 |
| 4 | 407 | 27.9 | 82.0 |
| 5 | 204 | 14.0 | 96.0 |
| 6 | 51 | 3.5 | 99.5 |
| 7 | 8 | 0.5 | 100.0 |
| Total | 1,460 | 100.0 |  |

***Table 57:*** *Missing food groups for Children by MDD status (6-23 months)*

|  | MDD | No MDD | 4 FG | 3 FG |
| --- | --- | --- | --- | --- |
|  | Percent | Percent | Percent | Percent |
| Eggs | 69.6 | 95.5 | 91.6 | 94.8 |
| Legumes and Nuts | 40.7 | 92.1 | 86.0 | 91.3 |
| Other Fruits/ Vegetables | 81.0 | 96.0 | 92.4 | 96.5 |
| Dairy | 54.8 | 93.0 | 90.9 | 89.5 |
| Flesh Foods | 4.2 | 50.2 | 9.6 | 41.3 |
| Vitamin A Fruits/ Vegetables | 10.6 | 58.8 | 17.2 | 57.8 |
| Breastmilk | 13.3 | 13.9 | 10.8 | 22.4 |
| Grains, Roots, Tubers | 0.4 | 17.1 | 1.5 | 6.4 |
| Observations | 263 | 1,197 | 407 | 344 |

MDD: Minimum Dietary Diversity, FG: Food Groups
